# Distinguishing between household headship with and without power and its association with subjective well-being among older adults: an analytical cross-sectional study in India

**DOI:** 10.1186/s12877-021-02256-0

**Published:** 2021-05-12

**Authors:** Shobhit Srivastava, S. K. Singh, Manish Kumar, T. Muhammad

**Affiliations:** 1 Department of Mathematical Demography and Statistics, International Institute for Population Sciences, Mumbai, Maharashtra 400088 India; 2 Department of Population Policies and Programs, International Institute for Population Sciences, Mumbai, Maharashtra 400088 India

**Keywords:** Household headship, Subjective wellbeing, Older adults, India

## Abstract

**Background:**

The status of household headship accorded to the older members of the family is often symbolic and seldom vested with some control over resources. The increased dependency and diminished ability to contribute to household economy are major factors that lead to a decline in the respect accorded to older people and their status in the family. The present study aimed to understand the distinction between the functional and nominal household headship status of older adults based on their decision-making power and examine how it is associated with their subjective well-being.

**Method:**

The present research used data from the 'Building a Knowledge Base on Population Aging in India' (BKPAI) which is nationally representative. The survey was conducted in 2011, across seven states of India. Descriptive statistics along with percentage distribution were calculated for subjective well-being over explanatory variables. For finding the association between subjective well-being over explanatory variables, binary logistic regression model was used.

**Results:**

The mean age of the study population was 68 years [CI: 67.8–68.2]. About 5 % of older adults had nominal while 95% had functional headship status. The prevalence of low subjective well-being (LSWB) was significantly higher among older adults with nominal headship status (58%) than functional headship status (23%). After controlling for several other variables, older adults with nominal headship status were 59% significantly more likely to have low subjective well-being than individuals with functional headship status (OR = 1.59; 95% CI: 1.10, 2.31). Further, older adults with psychological distress, chronic morbidity, poor self-reported health, no community involvement and no one to trust on were at higher risk of LSWB than their counterparts.

**Conclusions:**

Findings suggest that older adults who do not have a household headship with power with active participation in household decision-making as well as those who have no involvement in social activities or have poor health conditions need to be given more attention. Thus, to keep a large proportion of older population gainfully engaged, their care and support should be ensured via providing appropriate services that would enhance their roles and responsibilities and overall wellbeing.

## Introduction

According to census 2011, In India, 8 % of the total population were above the age of 60 years [1], and as per the estimates prepared by United Nations, it is likely to rise to 19% by 2050 [2]. Among a rapidly growing older population, a multitude of resource constraints contributes to loss of self-esteem and adverse psychological effects [3]. In the South Asian settings where family ties are considered paramount, intergenerational conflicts may have negative consequences on the well-being of older individuals [4].

The status of household headship accorded to the older members of the Indian families which is often symbolic and seldom vested with some control over resources is ambivalent in the existing literature. Study shows that increased dependency and diminished ability to contribute to household economy were found as major factors that lead to a decline in the respect accorded to older people and their status in the family [5]. While grown-up children tend to make decisions as they become the main breadwinners in the household, the decision-making power of older persons in India has been declining [6]. It is found that although they own private assets and share the same amount of intra-household transfers, older adults who reside with young and adult children were less likely to be household heads than were those with spouses and grandchildren [7]. Nevertheless, older adults often tend to maintain some control over their resources to prevent themselves from feeling like a burden to the family members and to retain children’s respect [8]. For this cause, they may actively involve in household decision-making and try to establish themselves as independent heads of the household [9].

As evidence suggests, if older individuals consider themselves as the household heads and their adult children stop taking their opinion into account for important household decisions, it can negatively affect their mental well-being [10]. Similarly, functional capacities are recognized as being shaped by class, gender, and other factors and functional old age can be delayed through the provision of adequate care and support [11, 12]. On the other hand, older adults depending on their children or family members to look after them who often consider them as a burden may result in ill-treatment and a multitude of health issues [13, 14]. Also, older members who take on fewer and lighter domestic duties, gain respect but experience a decline in their tangible household political and economic powers [15]. Hence, those older individuals who withdraw from active engagement in household activities after reaching a particular age are considered as transitioning from household headship to merely a senior member in the household and eventually giving up the material pleasure in their lifecycle [16]. Notably, evidence suggests that in order to promote active engagement of older adults, there is a need for an increased sense of coherence and personal autonomy [10, 17]. Thus, despite satisfaction with participation being a challenge to successful aging of the older population, it can also be a distinctive factor of actual participation and the quality of participation [18].

Furthermore, normal functioning that includes involvement in daily household activities is crucial to the well-being of older adults [16]. And with increased age, the likelihood that a person aged 60 and older will head his or her household increases [19]. However, the linkage between perceived statuses and functional support that is actually provided has been examined with less care. Besides, a headship status representing members’ shared interests is regarded as inadequate and inappropriate when it is automatically ascribed to the senior male’ [20]. Even though, when the value system becomes stronger and actual receipt of social supports are ensured, people generally become satisfied in old age and experience relatively high levels of emotional well-being [21]. Further, studies found gender differences in personal significance that a person attributes to the roles he or she occupies and the satisfaction from such role-making varies by its meanings [22]. Hence, with an unprecedented increase in the proportion of population over age 60 years in India that is expected to rise to nearly 20% by the year 2050 [23], it is important to better understand the conditions under which wellbeing of older adults can be ensured.

In this regard, we hypothesize that older adults who reported having a role in household decision-making were more likely to functionally head their households than their counterparts who often remain heads without any role-making. And the present study attempts to fill in a gap in well-being research in India by accessing data that explicitly have asked questions concerning decision-making in the household and examining the distinction between the functional and nominal household headship status of older adults based on their decision making power and how it is associated with their subjective well-being.

## Methods

### Data

The present research used data from Building a Knowledge Base on Population Aging in India (BKPAI) which is nationally representative. The survey was conducted in 2011, across seven states of India. The survey was sponsored by the Institute for Social and Economic Change (ISEC), Bangalore, Institute for economic growth (IEG), Delhi, Tata Institute for Social Sciences (TISS), Mumbai, and United Nations Population Fund (UNFPA), New Delhi [24]. The survey gathered information on various socio-economic, demographic, and health aspects of aging among households of those aged 60 years and above. The data from all the seven states were collected which represents the four regions of India. The states of Punjab and Himachal represent the northern part, West Bengal and Orissa represent the eastern part, Tamil Nadu and Kerala represent the southern part, and Maharashtra represents the western part of the country. The urban and rural samples within each state were drawn separately. The PSUs in the rural areas were villages, whereas the urban wards were the PSUs in the urban areas. First, villages were classified into different strata based on population size, and the number of PSUs to be selected was determined in proportion to the population size of each stratum. Using probability proportional to population size (PPS) technique, the primary sampling units (PSUs) were selected and within each selected PSU, elderly households were selected through systematic sampling [24]. A similar procedure was applied in drawing samples from urban areas. However, a total of 8329 households were interviewed and among them, 9852 older adults’ interviews were conducted [24]. The study only included those older adults who were the head of the households i.e. the effective sample size for the study was 4604 older adults.

### Variable description

#### Outcome variable

The outcome variable was subjective well-being among older adults. Nine questions were asked to assess this variable which includes a. Feels like life is interesting b. Compared with the past, it feels like present life is better c. On the whole, how happy with the kind of things doing in recent year’s d. Achieved the standard of living and the social status in life as expected e. The extent to which have achieved success and getting ahead f. Feels like normally accomplished whatever wanted to accomplish g. Feels like it is able to manage situations even when they do not turn out to be as expected h. Feels like confident that in case of crisis (anything that substantially upsets the situation in life), will be able to handle it or face it boldly i. With the things going on now, feel confident in coping with the future. The responses were 1 “Most of the time”, 2 “Sometimes”, and 3 “Hardly ever”. The responses were coded as 0 “most of the time/sometime” and 1 “hardly ever”. A scale of 0–9 was then generated using egen command in Stata-14 and was categorized as 0 “high” experiencing better experience (representing 6+ scores) and 1 “low” experiencing negative experience (representing score 5 and less) (Cronbach alpha: 0.89) [14, 25, 26].

#### Explanatory variable

1. The main explanatory variable was headship status among older adults i.e. whether the status was nominal or functional. The nominal headship was defined as the head who does not have any decision-making power in the household whereas the functional head was the head who has the absolute/partial power to make household decisions. The variable was generated using two variables i.e. first whether the older adult is the head of the household or not and whether he makes the major household decision or not. The sample only includes the older adults who were the heads of the household. The decision-making power was assessed using six questions which include “Who usually makes the following decisions: you alone or with your spouse, with your children, or with others?” on the following issues (a). Marriage of son/daughter. (b). Buying and selling of property (c). buying other household items (d). Gifts to daughters, grandchildren, other relatives (e). Education of children, grandchildren (f). arrangement of social and religious events (Cronbach alpha: 0.88). The responses were coded as 0 “no role in decision making” and 1 “full/partial role in decision making” i.e. decide alone or with your spouse, with your children, or with others. Headship status was coded as 0 “nominal head” which combines head with no role as decision-maker in the household and 1 “functional head” which combines head with full/partial role in decision-maker in the household.
2. Age was recoded as 60–69, 70–79, and 80 + years.
3. Sex was recoded as male and female.
4. Educational status was recoded as no schooling, below 5 years of schooling, 6–10 years of schooling, and 11 and above years of schooling [14].
5. Marital status was recoded as currently in union and not in union “included never married, widowed, divorced and separated”.
6. Co-residing with children was coded as “no” and “yes”
7. Working status was recoded as “working”, “not working” and “retired”. The status was for the last year [27].
8. Community involvement was generated using the following question a. attended a public meeting in the last 11 months with a discussion on local, community, or political affairs, b. Have attended any group, club, society, union, or organizational meeting in last 11 months, c. Have worked with other people in the neighbourhood to fix or improve something in the last 11 months, d. Have attended or participated in any religious programs/services etc. (not including weddings and funerals) in the last 11 months, and e. Have gone out of the house to visit friends or relatives in the last 11 months. The responses were never, rarely, occasionally, and frequently. They were coded as 0 “never” and 1 “rarely/occasionally/frequently” A scale of 0–5 was generated and was coded as 0 “no community involvement” and 1–4 were coded as 1 “community involvement” [28].
9. Trust over someone was assessed using the question “do you have someone you can trust and confide in?” which was recoded as 0 “yes” and 1 “no” [28].
10. Self-rated health had a scale of 1 to 5 “poor to excellent” and was recoded as 0 “good” (representing good, very good, and excellent) and 1 “poor” (representing poor or fair) [29].
11. Psychological distress was having a scale of 0 to 12 based on experiencing stressful symptoms and was recoded as 1 “high” (representing 6+ scores) and 0 “low” (representing score 5 and less) (Cronbach alpha: 0.90) [14, 25, 28]. The variable was coded using 12 questions namely a. Recently able to concentrate on whatever doing b. Recently lost much sleep due to some worry c. Recently felt constantly under strain d. Recently felt like couldn’t overcome difficulties e. Recently been feeling unhappy and depressed f. Recently been losing self-confidence g. Recently been thinking self as a worthless person h. Recently felt like playing a useful role in life i. Recently felt capable of making decisions about things j. Recently been able to enjoy normal day-to-day activities k. Recently been able to face up problems l. Recently been feeling reasonably happy, all things considered.
12. Chronic morbidity was recoded as 0 “no” and 1 “yes”. Twenty chronic diseases were used to generate variable chronic morbidity which includes Arthritis/rheumatism/Osteoarthritis, Diabetes, Asthma, Chronic lung disease (emphysema, bronchitis, COPD), etc.
13. Disability status was coded as 0 “no” and 1 “yes”. Disabilities included disability of vision, hearing, memory, walking, teeth (chewing), and speaking. Full and partial disability was clubbed as 1 “yes” and neither of any was clubbed as 0 “no”.
14. The wealth index drawn based on the BKPAI survey is based on the following 30 assets and housing characteristics: household electrification; drinking water source; type of toilet facility; type of house; cooking fuel; house ownership; ownership of a bank or post-office account; and ownership of a mattress, a pressure cooker, a chair, a cot/bed, a table, an electric fan, a radio/transistor, a black and white television, a colour television, a sewing machine, a mobile telephone, any landline phone, a computer, internet facility; a refrigerator, a watch or clock, a bicycle, a motorcycle or scooter, an animal-drawn cart, a car, a water pump, a thresher, and a tractor. The range of index was from poorest to the richest i.e. ranging from lowest to the highest [24]. The five categories of wealth are based in quintile i.e., lowest 20% to highest 20% (poorest, poorer, middle, richer and richest).
15. Caste was recoded as Scheduled Tribe, Scheduled Caste, Other Backward Class, and others [30]. The Scheduled Caste include “untouchables”; a group of the population that is socially segregated and financially/economically by their low status as per Hindu caste hierarchy. The Scheduled Castes (SCs) and Scheduled Tribes (STs) are among the most disadvantaged socio-economic groups in India. The OBC is the group of people who were identified as “educationally, economically and socially backward”. The OBC’s are considered low in the traditional caste hierarchy but are not considered untouchables. The “other” caste category is identified as having higher social status [30].
16. Religion was recoded as Hindu, Muslim, Sikh, and others
17. Place of residence was coded as urban and rural
18. Data for seven states was available in the data as mentioned in the data section [24].

### Statistical analysis

Descriptive statistics along with percentage distribution were calculated for subjective well-being over explanatory variables. Chi-square test [31] was used to find the significance level for the bivariate association between the outcome and the explanatory variables. For finding the association between subjective well-being over explanatory variables binary logistic regression model [32] was used. The outcome variable was subjective well-being coded as “high (0) and low (1)” and the main explanatory variable was headship status coded as “nominal” and “functional”.

The equation for logistic distribution is as follows:-

$$ \mathit{\ln}\left(\frac{\pi }{1-\pi}\right)={\beta}_0+{\beta}_1{X}_1+{\beta}_2{X}_2+{\beta}_3{X}_3\dots .{\beta}_n{X}_n $$

Where, *β*_0_, …. . , *β*_*M*_, are the regression coefficients indicating the relative effect of a particular explanatory variable on the outcome. These coefficients change as per the context in the analysis in the study. STATA 14 [33] was used for the analysis purpose.

## Results

Table 1 provides the socio-economic and demographic profile of the Indian older adults included in the analysis. The mean age of the study population was 68 years [CI: 67.8–68.2]. In the sample, about 5 % of older adults had nominal while 95% had functional headship status. More than half of the individuals (63%) belong to the age-group 60–69 years and nearly 10% were 80 years or older. Three-fourths (73%) of the older adults were male. Around 41% of the elderly were uneducated, and 62% had attained less than primary education. Two-third of the elderly (64%) were currently in a union and around 70% were co-residing with their children. One-third of the older adults (32%) were working, while 15% were retired at the time of the survey. Nearly 18% of the elderly reported no community involvement and around 17% reported that they do not trust someone. The health-related factors of the older adults were also included in the analysis. More than half of the elderly (53%) reported poor health status, and nearly 20% had a high level of psychological distress. Nearly 62% of older adults had chronic morbidity, and around 70% were disabled. According to religion, the majority of respondents were Hindus (80%). Nearly 72% of the older adults were rural residents.

**Table 1:** Socio-economic and demographic profile of the study population in India

Background characteristics	Sample	Percentage
**Headship status**		
Nominal	207	4.5
Functional	4397	95.5
**Age group (years)**		
60–69	2903	63.1
70–79	1254	27.2
80+	447	9.7
**Sex**		
Male	3342	72.6
Female	1262	27.4
**Educational attainment**		
Not educated	1876	40.7
5 years or less	985	21.4
6–10 years	1381	30.0
11+ years	362	7.9
**Marital status**		
Not in union	1670	36.3
Currently in union	2934	63.7
**Children co-residing**		
No	1381	30.0
Yes	3223	70.0
**Working status (last 1 year)**		
Not working	2504	54.4
Working	1476	32.1
Retired	624	14.6
**Community involvement**		
No	837	18.2
Yes	3767	81.8
**Trust over someone**		
No	788	17.1
yes	3816	82.9
**Self-rated health**		
Good	2137	46.4
Poor	2467	53.6
**Psychological distress**		
Low	3667	79.6
High	937	20.4
**Chronic morbidity**		
No	1748	38.0
Yes	2856	62.0
**Disability**		
No	1382	30.0
Yes	3222	70.0
**Wealth status**		
Poorest	1066	23.2
Poorer	1005	21.8
Middle	972	21.1
Richer	873	19.0
Richest	686	14.9
**Religion**		
Hindu	3650	79.3
Muslim	327	7.1
Sikh	423	9.2
Others	203	4.4
**Caste**		
Scheduled Tribe	981	21.3
Scheduled Caste	225	4.9
Other Backward Class	1726	37.5
Others	1673	36.3
**Type of residence**		
Rural	3298	71.6
Urban	1306	28.4
**State**		
Himachal Pradesh	738	16.0
Punjab	649	14.1
West Bengal	584	12.7
Orissa	544	11.8
Maharashtra	660	14.3
Kerala	636	13.8
Tamil Nadu	792	17.2
**Total**	4604	100.0

### Bivariate analysis

The bivariate analysis of LSWB by various socio-economic and demographic characteristics is presented in Table 2. The results suggest the significant bivariate associations between LSWB and all the selected background characteristics included in the analysis. The prevalence of LSWB was significantly higher among older adults with nominal headship status (58%) than functional headship status (23%). The proportion of older adults with LSWB increases with an increase in age-groups. The prevalence of LSWB was found significantly higher among females, uneducated, separated or widowed, and non-working older adults than their respective counterparts. According to health status, the LSWB was more prevalent among older adults with chronic morbidity, psychological distress, poor self-rated health, and disability. The older adults with the poorest wealth status (45%) and rural place of residence (25%) reported higher LSWB. According to various Indian states, the prevalence of LSWB was reportedly highest in West Bengal (48%), followed by Maharashtra (34%) and Tamil Nadu (33%).

**Table 2:** Percentage of low subjective well-being by background characteristics among older adults in India

Background characteristics	LSWB (%)	Chi-square ***p***-value
**Headship status**		0.001
Nominal	58.0	
Functional	22.8	
**Age group (years)**		0.001
60–69	22.1	
70–79	26.3	
80+	33.4	
**Sex**		0.001
Male	22.0	
Female	30.5	
**Educational attainment**		0.001
Not educated	33.7	
5 years or less	25.9	
6–10 years	13.7	
11+ years	12.0	
**Marital status**		0.001
Not in union	30.0	
Currently in union	21.1	
**Children co-residing**		0.001
No	29.5	
Yes	22.1	
**Working status (last 1 year)**		0.001
Not working	31.0	
Working	20.5	
Retired	6.7	
**Community involvement**		0.001
No	37.7	
Yes	21.4	
**Trust over someone**		0.001
No	41.6	
yes	20.8	
**Self-rated health**		0.001
Good	12.4	
Poor	34.6	
**Psychological distress**		0.001
Low	15.2	
High	60.0	
**Chronic morbidity**		0.001
No	21.0	
Yes	26.4	
**Disability**		0.001
No	14.8	
Yes	28.4	
**Wealth status**		0.001
Poorest	44.7	
Poorer	31.7	
Middle	17.4	
Richer	11.8	
Richest	7.7	
**Religion**		0.001
Hindu	26.1	
Muslim	26.5	
Sikh	8.8	
Others	22.4	
**Caste**		0.001
Scheduled Tribe	31.0	
Scheduled Caste	28.5	
Other Backward Class	25.2	
Others	19.0	
**Type of residence**		0.001
Rural	25.1	
Urban	22.5	
**State**		0.001
Himachal Pradesh	11.0	
Punjab	9.5	
West Bengal	47.8	
Orissa	26.8	
Maharashtra	33.6	
Kerala	11.5	
Tamil Nadu	32.5	
Total	24.3	

### Multivariate analysis

Table 3 summarises the adjusted odds ratio estimates for low subjective well-being (LSWB) by background characteristics of Indian older adults. After controlling for various other variables, older adults with nominal headship status were 1.59 times significantly more likely to have LSWB than individuals with functional headship status (OR = 1.59; 95% CI: 1.10, 2.31). The individuals aged 80 years and above had 34% significantly higher odds of having LSWB compared to the individuals belonging to the age group 60 to 69 years (OR = 1.34; 95% CI: 1.01, 1.79). According to educational attainment, older adults with no or less than the primary level of education had significantly higher odds of having LSWB than those with more than 11 years of education. We did not find any association between marital status and LSWB among older adults. Results further showed that the LSWB among older adults is not associated with their status of living with children. Non-working older adults were found to have 30% significantly higher odds of LSWB than working older adults (OR = 1.30; 95% CI: 1.06, 1.61).

**Table 3:** Logistic regression estimates for low subjective well-being by background characteristics among older adults in India

Background characteristics	LSWB AOR (95%CI)
**Headship status**	
Nominal	1.59*(1.10,2.31)
Functional	Ref.
**Age group (years)**	
60–69	Ref.
70–79	1.05 (0.87,1.28)
80+	1.34*(1.01,1.79)
**Sex**	
Male	Ref.
Female	0.95 (0.70,1.27)
**Educational attainment**	
Not educated	1.83*(1.2,2.78)
5 years or less	1.71*(1.13,2.59)
6–10 years	1.15 (0.77,1.71)
11+ years	Ref.
**Marital status**	
Not in union	Ref.
Currently in union	0.99 (0.75,1.3)
**Children co-residing**	
No	Ref.
Yes	1.00 (0.82,1.21)
**Working status (last 1 year)**	
Not working	1.30*(1.06,1.61)
Working	Ref.
Retired	0.770.53, 1.12)
**Community involvement**	
No	1.49*(1.20,1.84)
Yes	Ref.
**Trust over someone**	
No	1.72*(1.38,2.15)
yes	Ref.
**Self-rated health**	
Good	
Poor	2.09*(1.73,2.52)
**Psychological distress**	
Low	Ref.
High	5.60*(4.64,6.76)
**Chronic morbidity**	
No	Ref.
Yes	1.21*(1.00,1.47)
**Disability**	
No	Ref.
Yes	1.60*(1.28,1.99)
**Wealth status**	
Poorest	3.13*(2.14,4.58)
Poorer	2.34*(1.66,3.32)
Middle	1.71*(1.22,2.39)
Richer	1.45*(1.03,2.03)
Richest	Ref.
**Religion**	
Hindu	Ref.
Muslim	1.18 (0.85,1.64)
Sikh	0.87 (0.50,1.52)
Others	1.14 (0.73,1.77)
**Caste**	
Scheduled Tribe	Ref.
Scheduled Caste	0.84 (0.55,1.27)
Other Backward Class	1.00 (0.78,1.28)
Others	0.92 (0.72,1.17)
**Type of residence**	
Rural	Ref.
Urban	1.39*(1.15,1.69)

The analysis is controlled for states also; *Ref* Reference, *AOR* Adjusted Odds ratio, *CI* Confidence interval

*if *p* < 0.05

Older adults with psychological distress, chronic morbidity, poor self-reported health, no community involvement and no one trust to on were at comparatively higher risk of LSWB than their counterparts. According to the wealth index, the odds of having LSBW increases with a decline in wealth index; for instance, the older adults in the poorest category do have almost three times significantly higher odds of having LSBW compared to older adults in the richest category (OR = 3.13; 95% CI: 2.14, 4.58). The older adults’ who resided in the urban area had nearly 39% significantly higher likelihood of having LSWB compared to their rural counterparts (OR = 1.39; 95% CI: 1.15, 1.69). We did not find any association of LSWB with religion and caste.

Table 4 represents the stratified analysis by gender. It was found that older males who were nominal heads had a 60% significantly higher likelihood to suffer from LSWB than the older males who were functional heads [AOR: 1.60; 95% CI: 1.33–2.93]. Similarly, older females who were nominal head had a 69% significantly higher likelihood to suffer from LSWB than the older females who were functional head [AOR: 1.69; 95% CI: 1.03–2.79].

**Table 4:** Logistic regression estimates for low subjective well-being by sex among older adults in India

Background characteristics	LSWB AOR (95%CI)	
	Male	Female
**Headship status**		
Nominal	1.60*(1.33–2.93)	1.69*(1.03–2.79)
Functional	Ref.	Ref.

The analysis was controlled for the other factors that were presented in table-3; Additionally, the analysis is controlled for states; *Ref* Reference, *AOR* Adjusted Odds ratio, *CI* Confidence interval *if *p* < 0.05

## Discussion

In order to determine the major factors associated with the level of subjective well-being of older individuals especially their actual headship status, a binary logistic regression was employed and it has shown statistically significant associations. Older people after a certain age consider themselves as physically aged and mentally withdraw from the roles and responsibilities they feel they are unable to perform [19]. Such a withdrawal itself reinforces the feelings of sickness and weakness and results in a decline in their overall well-being [16]. Consistently, the older adults who were nominal heads with no role in household decision making in the current study were more likely to report lower levels of subjective wellbeing. This finding suggests that policies and interventions can create more opportunities for meaningful engagement of older individuals and establish a more age-friendly household environment with an ultimate goal of promoting their late-life wellbeing. Also, the older parents should be enabled to become more actively involved in household activities and strengthen the intergenerational relationships.

Other findings of the present study suggest that several socio-demographic factors including age, level of education, community involvement, and trust were significant predictors of subjective well-being in old age. Age was found a significant predictor of subjective well-being among older Indian adults and it shows that with increasing age, subjective well-being will decrease. On the whole, the finding is consistent with and supports current wellbeing literature [34–36]. A possible explanation for the negative effects of age on subjective well-being may be the result of life stresses, such as widowhood, poor health condition, the decline in social and family roles, and decline in social engagement. Consistent with previous studies, older adults who were involved in community activities or had trust in someone reported a higher level of subjective well-being than their counterparts [28]. It is believed that social support is a powerful source of emotional wellbeing that results in higher levels of overall well-being especially in traditional societies [37, 38]. Similarly, older people who are socially active have a better chance of benefiting from interpersonal relationships and suffer less from loneliness [39, 40]. While being in a marital union was significantly associated with subjective well-being at a bivariate level, it was not a predictor in the multivariable analyses.

Education has always been hailed as an essential factor of wellbeing in late life. It has been linked with better opportunities, better health, and a high standard of living [41]. Also, a vast proportion of the older Indian adults is uneducated and thereby unskilled and is mostly engaged in the unorganized sector leading to higher dependency on their children at old age and to a lower status [42]. The same is reflected in the current regression results where the likelihood of low well-being increases with a decrease in educational status. Illiterates and older individuals with primary schooling are likely to be of low well-being with reference to highly educated and it is statistically significant. The finding suggests the importance of higher education that leads to better awareness as well as better economic opportunities thereby ensuring higher levels of well-being in older ages [43]. The implications are particularly relevant in an Indian socio-cultural setting where people are oftentimes classified as old in relation to their inability to perform roles and responsibilities [16].

The positive association of psychological distress with LSWB observed in our study can be explained as the psychological distress may reflect the differences in health conditions and issues of access to resources and mental health care [44]. As evidence suggests physical health status plays a preponderant role in late-life wellbeing [45–47]. Consistently, the present study found a significant positive association of poor self-rated health and prevalence of chronic morbidity with LSWB. The finding that disability had a significant positive association with LSWB was in concordance with earlier studies that highlighted functional activities namely, activities of daily living (ADLs), instrumental ADLs as predictors of subjective well-being in later years of life [17, 18]. This also supports the findings that reduced physical functionality among older adults is related to poor mental wellbeing [48, 49].

In addition, household wealth status appeared to be an important factor associated with subjective well-being among older Indian adults. This finding is consistent with some previous studies which have found that household economic status is a significant predictor of quality of life and psychological well-being [50–52]. Also it supports the notion that people from higher wealth quintiles can easily satisfy their basic needs such as food, housing, and health; therefore, a higher level of well-being is attained. Finally, several studies have found rural–urban differences in terms of psychological well-being, quality of life, life satisfaction, depression, happiness, and mental health among elderly people [53, 54]. The finding of our study that indicated that urban place of residence as a positive factor of subjective wellbeing in old age can be explained by the differentials in rural-urban lifestyles and the highly available social networks in urban areas.

As with any study, there are several limitations to this study to be acknowledged. The first is the cross-sectional nature of this study which prevents the possibility of drawing conclusions about causal relationships between the variables studied. Second, although this study had a large sample size, since it was carried out among older individuals in seven states of India, there should be caution while results being generalized to the older population across the country.

## Conclusion

Policy makers should pay special attention to the vulnerable groups of older population and promote interventions according to their needs. In addition, older adults who do not have a household headship with power with active participation in decision making as well as those who have no involvement in social activities or have poor health conditions need to be given more attention. Thus, to keep a large proportion of older population gainfully engaged, their care and support should be ensured via providing appropriate services that would enhance their roles and responsibilities and overall wellbeing.

## Data Availability

The study utilizes a secondary data which is available only on request from director@isec.ac.in or india.office@unfpa.org.

## Abbreviations

LSWB: Low Subjective well-being
CI: Confidence interval
AOR: Adjusted Odds ratio
UNFPA: United Nation’s Population Fund
BKPAI: Building a Knowledge Base on Population Aging in India
PSU: Primary sampling unit
